# Quantitative Facet Joint Effusion on Magnetic Resonance Imaging Is Associated With Dynamic Segmental Instability and Pain Severity in Degenerative Lumbar Spondylolisthesis

**DOI:** 10.14740/jocmr6572

**Published:** 2026-06-30

**Authors:** Yu Lin Zhao, Hai Peng Si, Yong Guang Sun

**Affiliations:** a Department of Orthopedics, Qilu Hospital of Shandong University (Qingdao), Cheeloo College of Medicine, Shandong University, Qingdao, Shandong 266035, China; b Department of Orthopedics, Qilu Hospital of Shandong University, Cheeloo College of Medicine, Shandong University, Jinan, Shandong 250012, China; c Key Laboratory of Qingdao in Medicine and Engineering, Department of Orthopedics, Qilu Hospital (Qingdao), Shandong University, Qingdao, Shandong 266035, China; d Department of Surgery, Qilu Hospital of Shandong University (Qingdao), Cheeloo College of Medicine, Shandong University, Qingdao, Shandong 266035, China

**Keywords:** Facet joint effusion, Facet joint degeneration, MRI, Lumbar spine instability, Degenerative lumbar spondylolisthesis

## Abstract

**Background:**

The aims of the study were to determine whether quantitatively measured facet joint effusion (FJE) on magnetic resonance imaging (MRI) is associated with dynamic segmental instability and greater pain severity in patients with degenerative lumbar spondylolisthesis (DLS), and to evaluate the diagnostic performance of FJE for identifying unstable segments.

**Methods:**

This retrospective single-center study reviewed 151 consecutive DLS patients treated at Qilu Hospital (Qingdao) between January 1, 2020 and June 30, 2024. The cohort included 31 men and 118 women (mean age 64.52 ± 9.76 years). Slipped levels comprised L3 (n = 7), L4 (n = 116), and L5 (n = 28). Patients with prior lumbar surgery, acute spinal trauma, tumor, ankylosing spondylitis, marked scoliosis, multilevel spondylolisthesis, or retrolisthesis were excluded. On axial T2-weighted MRI, the maximal unilateral and bilateral facet effusion thicknesses were measured in millimeters. Dynamic imaging was used to classify segments as stable or unstable. Pain was quantified using the Visual Analog Scale (VAS). Statistical analyses compared FJE presence and size between stable and unstable segments and assessed diagnostic accuracy (sensitivity, specificity, receiver operating characteristic area under the curve) and correlations with slip distance and VAS.

**Results:**

Seventy-two patients (47.7%) had dynamic segmental instability. FJE was present at 93.1% of unstable levels, with mean effusion thickness of 2.75 ± 0.89 mm, whereas stable levels showed a 26.6% FJE incidence and mean thickness of 1.25 ± 1.25 mm (P < 0.05). Using presence of FJE to identify instability yielded sensitivity 93.1% and specificity 73.4%. Among 88 patients with effusion ≥ 1.0 mm, 76.1% exhibited instability. Effusion thickness correlated linearly with the anteroposterior slip distance difference. Facet joint degeneration grade related nonlinearly to effusion width (increase from grade 1 to 2, decline at grade 3; P < 0.05). ROC AUCs for left and right effusion thickness were 0.9243 and 0.9296, respectively (P < 0.0001). Patients with effusion had higher VAS scores (4.30 ± 1.07 vs. 2.79 ± 0.68, P < 0.05).

**Conclusions:**

Millimeter-quantified FJE on MRI was strongly associated with dynamic segmental instability and greater pain severity in DLS. Quantitative FJE demonstrated high diagnostic accuracy and may serve as a practical imaging marker to identify unstable lumbar segments. Given the retrospective design, these results indicate association rather than causation and require prospective validation.

## Introduction

Degenerative lumbar spondylolisthesis (DLS) refers to the anterior displacement of a lumbar vertebral body relative to the subjacent vertebra in the sagittal plane, with the posterior elements remaining intact. This condition is highly prevalent among middle-aged and elderly populations and represents a common etiology of lower back pain, radicular pain, and intermittent claudication [1]. The primary etiology of symptomatic manifestation is attributed to either instability at the slipped segment or neural compression resulting from spinal canal stenosis. In cases complicated by spinal canal stenosis causing sciatica and neurogenic claudication, surgical intervention may be warranted.

The preoperative assessment of the stability of the slipped lumbar segment holds significant value in guiding the formulation of the surgical plan for lumbar degenerative diseases [2, 3]. It is widely accepted that standing flexion-extension lumbar radiographs constitute the optimal modality for detecting the presence of dynamic spinal instability [4]. Magnetic resonance imaging (MRI) is generally the preferred diagnostic tool for evaluating patients presenting with neurogenic claudication and radiculopathy of the lower extremities [5]. MRI excels in identifying lumbar degenerative changes, including intervertebral disc degeneration, spinal canal stenosis, and facet joint degeneration (FJD)/arthritis/facet joint cysts. However, in numerous cases, supine MRI may fail to detect lumbar spine instability (LSI), with some studies reporting a missed diagnosis rate as high as 28% [6, 7]. This discrepancy is largely attributed to biomechanics under axial loading, where dynamic LSI is most conspicuously demonstrated in the upright position, whether by upright radiography or upright MRI [5, 7]. Nevertheless, MRI is most commonly performed in the supine position, and without concomitant dynamic radiographs, there exists a potential risk of underdiagnosis.

The presence of fluid within the facet joint is indicative of synovial FJD. Similar to effusions observed in other synovial joints affected by arthritis, such as the knee, shoulder, and hip, facet joint effusion (FJE) can be identified through MRI [8]. Compared to T1-weighted MRI sequences (commonly referred to as “fat sequences”), T2-weighted sequences depict extracellular free water with high signal intensity, whereas T1-weighted sequences are particularly adept at delineating normal anatomical structures. Consequently, T2-weighted sequences are most advantageous for detecting FJE. On supine MRI, in axial sections, FJE appears as a curvilinear high signal intensity within the facet joint, with signal characteristics comparable to cerebrospinal fluid on T2-weighted images. Previous biomechanical studies have underscored the importance of facet joint integrity in lumbar spine stability. It is logically consistent that lumbar segments exhibiting degenerative, fluid-filled facet joints demonstrate instability [9]. However, the relationship between FJE detected on MRI, radiographic LSI, and clinical manifestations such as low back pain (LBP) remains inconclusive and lacks consensus in the current literature.

The objective of this study is to analyze the relationship between FJE detected on MRI and sagittal plane instability detected on flexion-extension lumbar X-rays in patients with degenerative lumbar spine disease. Standing dynamic X-rays were used to definitively determine LSI in patients with DLS, and the correlation between LSI and FJE on supine sagittal T2W MRI was observed.

## Materials and Methods

### General information

A retrospective collection was conducted on the clinical and imaging data of 151 patients with DLS who were hospitalized in the Spine Surgery Department of Qilu Hospital of Shandong University (Qingdao) from January 1, 2020, to June 30, 2024. Among them, there were 31 males and 118 females; the average age was 64.52 ± 9.76 years (range, 45–87 years). The slipped segments included L3 in seven cases, L4 in 116 cases, and L5 in 28 cases. According to the Meyerding classification, there were 126 cases of grade I slippage, 24 cases of grade II, and one case of grade III. All patients had varying degrees of low back and leg pain symptoms, and the Visual Analog Scale (VAS) scores for LBP were collected.

Standing flexion-extension (F-E) lateral X-rays and supine MRI scans were obtained, all performed at our institution to ensure standardized imaging protocols. The distance between the patient and X-ray source was consistent, and the images were required to be clear with well-demonstrated vertebral endplates. Routine standing F-E lateral X-rays were obtained. This study is retrospective, and all examinations were part of routine clinical care without any additional tests or expenses due to this study.

Exclusion criteria were: (1) history of lumbar spine surgery, (2) acute spinal trauma, (3) lumbar spine tumor, (4) ankylosing spondylitis, (5) scoliosis, (6) multi-segment spondylolisthesis, and (7) lumbar retrolisthesis.

### Methods

**Figure 1:**
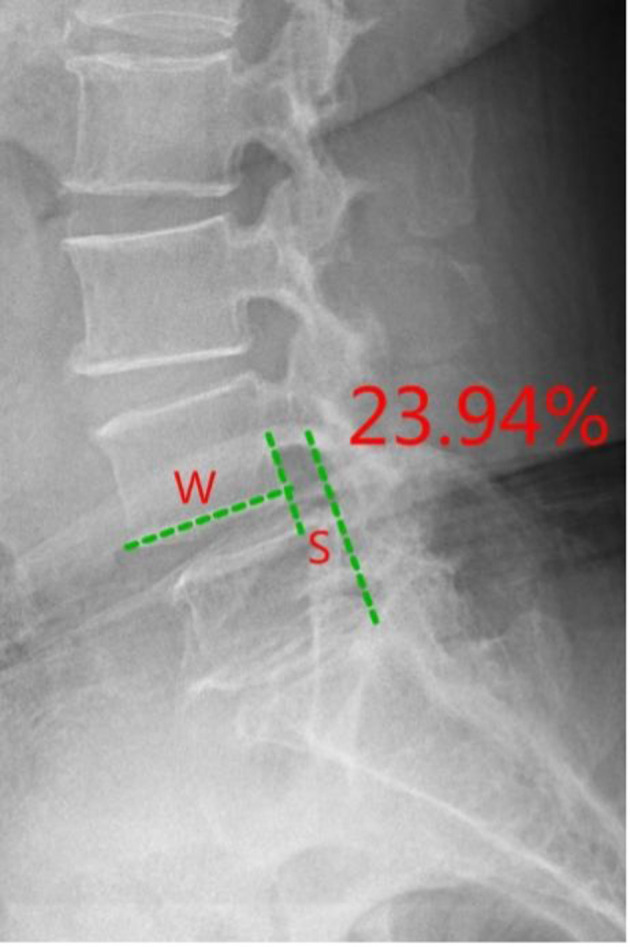
Lumbar spondylolisthesis ratio measurement.

Retrospective analysis of imaging data was performed using the Picture Archiving and Communication System (PACS). The imaging data were routinely collected upon admission for hospitalized patients, including standing flexion (F), extension (E) lateral X-rays, and supine MRI (S), with axial MRI images selected for analysis. The distance of lumbar spondylolisthesis and the width of the vertebral body at the slipped segment were measured using the method proposed by Dupuis et al [10] (Fig. 1). The percentage of slippage distance relative to vertebral body width was used for the final analysis. We calculated the difference in slippage percentage between standing flexion (F) and extension (E) positions, defined as the slip rate, which reflects the degree of anterior-posterior horizontal displacement at the slipped segment, serving as an indicator of segmental stability (Fig. 1). A slip rate ≥ 8% was defined as segmental instability [11]. This measurement method, compared to simply measuring the slippage value, eliminates the magnification differences caused by variations in equipment or patient-to-device distance, facilitating comparison between different radiologic images. For the study participants, LBP and leg pain VAS scores were collected to observe the relationship between LBP and leg pain.

**Figure 2:**
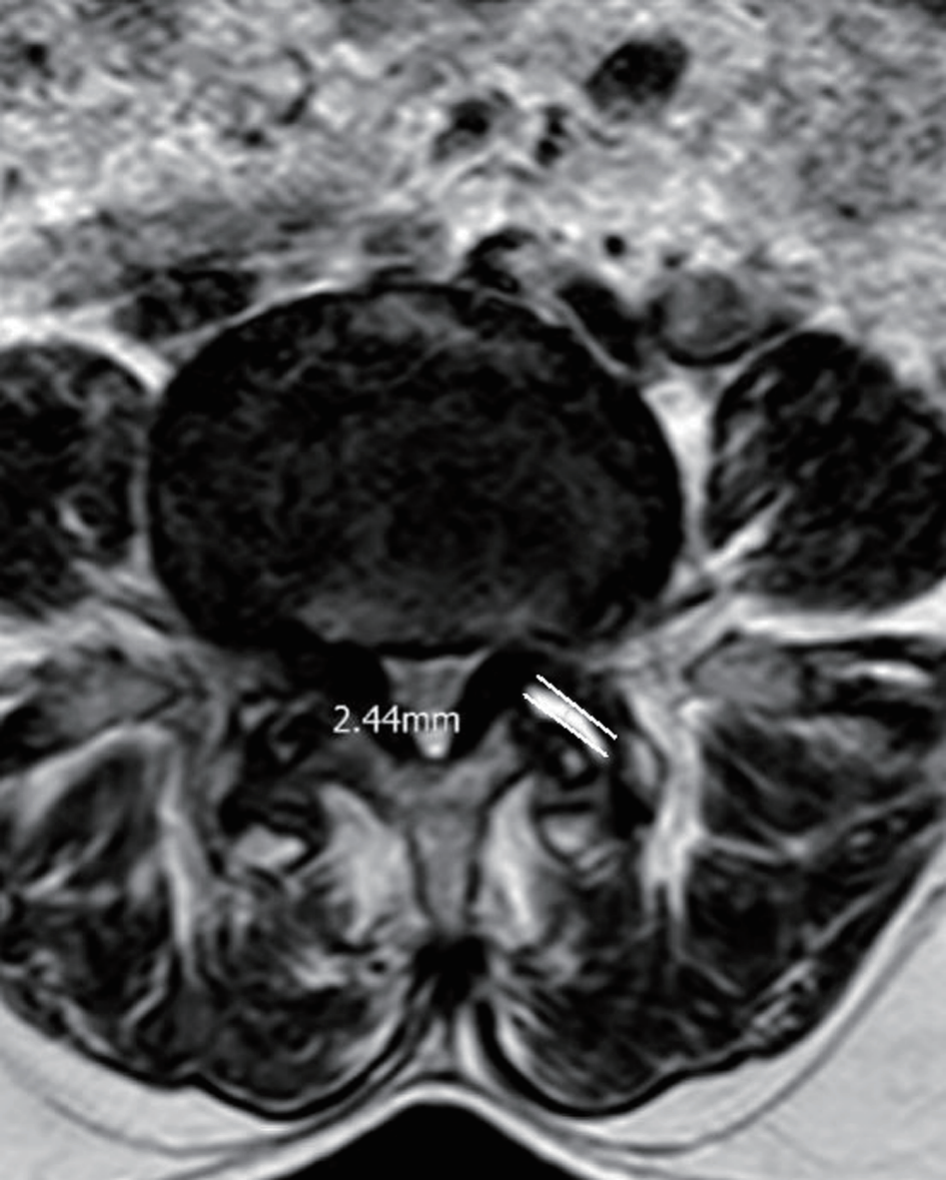
Measurement of small joint effusion. Measurement of facet joint effusion width on axial T2-weighted MRI. The axial slice showing the largest facet joint space was selected and the maximal anteroposterior distance between opposing articular surfaces (white line/arrow) was measured perpendicular to the joint plane. The measured distance is reported as the maximum facet joint effusion width (mm). MRI: magnetic resonance imaging.

Measurement of FJE was performed on routine axial T2-weighted MR images. FJE was defined as a focal, high-signal collection within the facet joint with signal intensity comparable to cerebrospinal fluid on T2. For each facet joint, we selected the axial slice that displayed the maximal joint space and measured the maximal anteroposterior distance between the opposing articular surfaces along a line perpendicular to the joint plane [8], This value was recorded as the maximum FJE width (mm) (Fig. 2). Because measurement was one-dimensional on axial images, the term “width” is used throughout rather than “volume.”

FJD was assessed using the widely accepted Weishaupt grading system [12]. Based on computed tomography (CT) and MRI findings of the facet joints, degeneration was classified into four grades: grade 0 was defined as a normal facet joint space (2 mm); grade 1 showed a narrowed joint space (< 2 mm) with or without facet hypertrophy and/or mild osteophyte formation; grade 2 was characterized by a narrowed joint space, moderate facet hypertrophy and/or moderate osteophyte formation, and minimal subchondral bone erosion; grade 3 involved a narrowed joint space (< 2 mm), severe facet hypertrophy and/or large osteophyte formation, severe subchondral bone erosion, and/or presence of subchondral cysts (Table 1).

**Table 1:** Grade of Lumbar Facet Joint Degeneration

Grade	Criteria
0	Normal facet joint width (2–4 mm)
1	Facet joint space narrowing, small osteophytes, and/or mild articular process hypertrophy
2	Facet joint space narrowing, moderate osteophytes, moderate articular process hypertrophy, and/or small subarticular bone erosions
3	Facet joint space narrowing, large osteophytes, severe articular process hypertrophy, subarticular bone erosions, and/or subchondral cyst formation

This study was approved by the Ethics Committee of Qilu Hospital (KYLL-KS-2024196) in accordance with the principles of the Declaration of Helsinki. Exemption from informed consent application has been obtained. Measurements were independently performed by two authors. To assess measurement reliability, we performed an inter-rater analysis. A random sample of segments (n = 30) was independently measured by two readers. Both readers were blinded to clinical information and to each other’s measurements. For the continuous measurement of maximum FJE width (mm), we calculated the intraclass correlation coefficient (ICC; two-way mixed effects, absolute agreement) with 95% confidence intervals (CIs) [13]. For the binary classification of effusion presence/absence, we calculated Cohen’s kappa with 95% CIs [14]. In the reliability sample, the ICC for maximum FJE width was 0.921 (95% CI 0.879–0.978), indicating excellent reliability; Cohen’s kappa for presence/absence of effusion was 0.78 (95% CI 0.65–0.91), indicating substantial agreement.

### Statistical analysis

All statistical analyses were performed using SPSS version 26.0 (IBM Corp., Armonk, NY, USA). Continuous variables were assessed for normality using the Kolmogorov–Smirnov test and are presented as mean ± standard deviation (SD) when approximately normally distributed or as median (interquartile range (IQR)) when non-normally distributed. Categorical variables are presented as counts and percentages.

Between-group comparisons were performed with the independent samples *t*-test for normally distributed continuous variables and the Mann–Whitney U test for non-normally distributed continuous variables. Categorical variables were compared using Pearson’s χ^2^ test or Fisher’s exact test when expected cell counts were small. Correlations between FJE measurements and lumbar segmental instability (LSI) parameters (e.g., slip percentage difference) were assessed using Pearson or Spearman correlation coefficients, as appropriate.

The diagnostic performance of FJE for predicting LSI was evaluated using receiver operating characteristic (ROC) curve analysis; area under the curve (AUC) with 95% CI and sensitivity and specificity at the optimal cutoff (Youden index) are reported. To examine independent associations with instability (dependent variable: LSI; unstable = 1, stable = 0), multivariable binary logistic regression was performed including maximum FJE (max FJE, continuous, mm) and the following prespecified covariates: sex, age, body mass index (BMI), hypertension, diabetes, smoking, and alcohol. Regression coefficients, odds ratios (ORs), and 95% CIs are reported. Model diagnostics included assessment of linearity in the logit for continuous predictors, multicollinearity (variance inflation factor), and model fit and discrimination (Hosmer–Lemeshow test and AUC). All tests were two-sided, and P < 0.05 was considered statistically significant.

## Results

### Result 1

**Figure 3:**
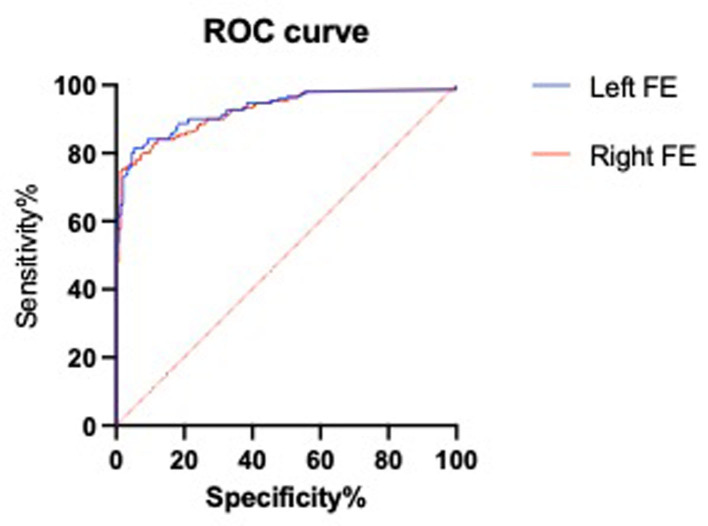
ROC curves of facet joint effusion (FJE) width for predicting lumbar segmental instability. Left AUC = 0.9243; Right AUC = 0.9296 (P < 0.0001). AUC: area under the curve; ROC: receiver operating characteristic.

**Figure 4:**
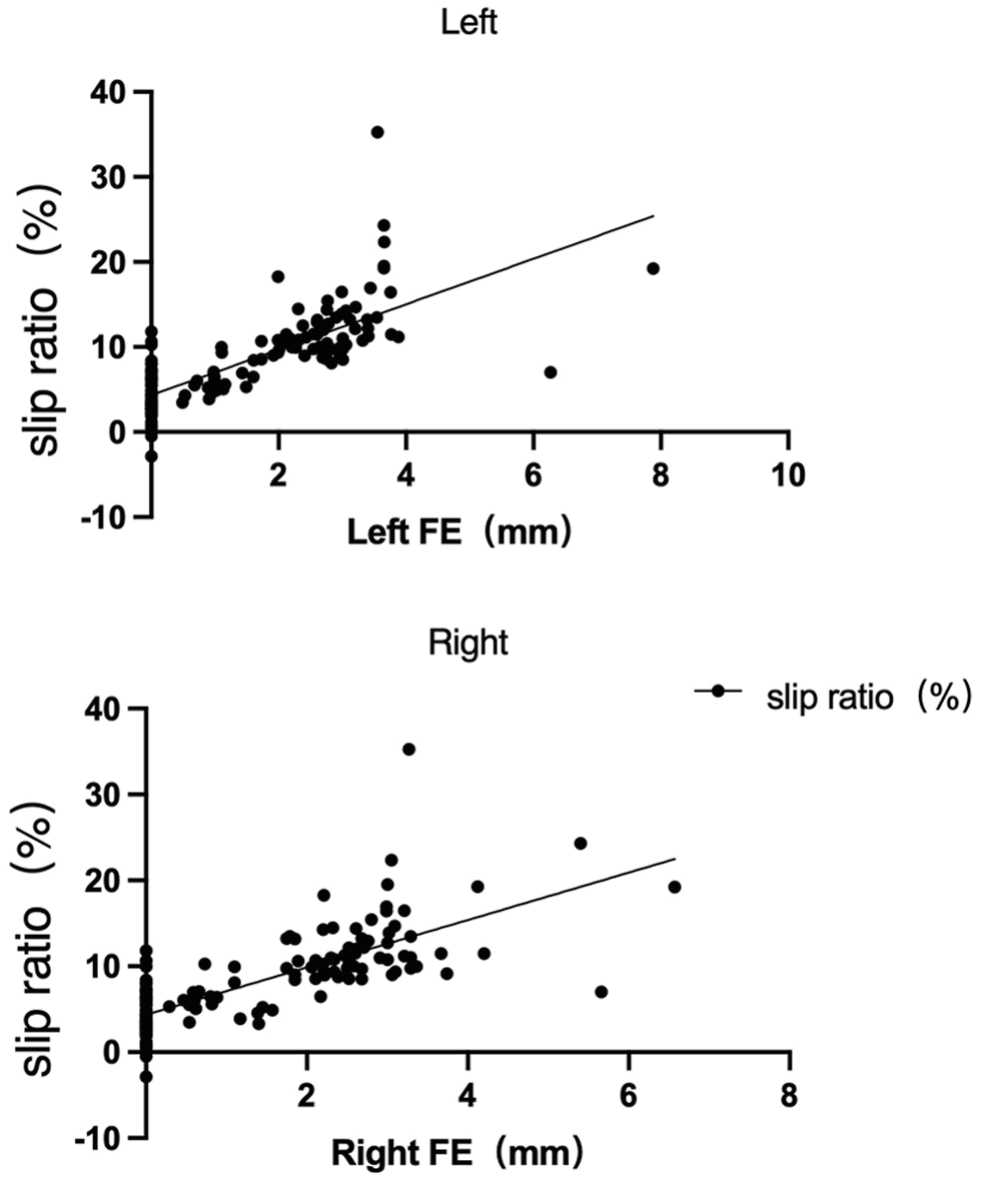
Linear regression of slip ratio against facet joint effusion (FJE) width for the left and right sides. Fitted equations: Left, Y = 2.673X + 4.339; Right, Y = 2.759X + 4.357.

A total of 151 patients were included in the analysis (72 unstable, 79 stable by slip rate ≥ 8%). Clinical background variables including age, sex, BMI, hypertension, diabetes, smoking, and alcohol use were recorded for all subjects and are summarized in the baseline table. Maximum FJE (max FJE, mm) was closely associated with LSI: 67 of 72 unstable patients (93.1%) had FJE at the index segment (65 bilateral, two unilateral), with mean FJE 2.75 ± 0.89 mm (left, n = 66: 2.80 ± 0.90 mm; right, n = 66: 2.70 ± 0.88 mm). Among 79 stable patients, 21 had FJE (14 bilateral, seven unilateral) and 58 (73.4%) had no effusion; mean FJE in the stable group was 1.25 ± 1.25 mm (left, n = 17: 1.31 ± 1.32 mm; right, n = 18: 1.20 ± 1.22 mm). The presence of FJE was strongly associated with instability (93.1% vs. 26.6%; Fisher’s exact, P < 0.001), with an unadjusted OR of 37.0 (95% CI 13.1–104.3). ROC analysis demonstrated excellent discrimination (AUC left = 0.9243, AUC right = 0.9296; P < 0.0001), sensitivity 93.1%, and specificity 73.4%; using thresholds, FJE ≥ 1 mm identified 78 patients (65/78 unstable, 83.3%) and FJE > 2 mm identified 65 patients (63/65 unstable, 96.9%). Importantly, after adjustment for potential confounders (sex, age, BMI, hypertension, diabetes, smoking, and alcohol) in a multivariable binary logistic regression model, max FJE remained an independent predictor of LSI (regression coefficient 1.825, SE 0.268; adjusted OR per 1 mm = 6.20, 95% CI 3.67–10.48, P < 0.001), while the listed covariates were not statistically significant in the adjusted model (all P > 0.05) (Tables 2 and 3). Model diagnostics (linearity in the logit for continuous predictors, multicollinearity, goodness of fit, and discrimination) supported the validity of the fitted model. Finally, the amount of FJE showed a linear relationship with the difference in slip percentage between lumbar flexion and extension (Figs 3 and 4; Table 4).

**Table 2:** Baseline Characteristics of the Study Population by Lumbar Segmental Instability Status

Variable	Total (n = 151)	LSI unstable (n = 72)	LSI stable (n = 79)	P value
Age, years	64.52 ± 9.76	63.81 ± 8.47	65.16 ± 10.81	0.372
Sex, male, n (%)	33/151 (21.85%)	13/72 (18.06%)	20/79 (25.32%)	0.330
BMI, kg/m^2^, mean ± SD	26.33 ± 4.20	27.42 ± 4.19	25.35 ± 4.00	0.002
Hypertension, n (%)	61/151 (40.40%)	36/72 (50%)	25/79 (31.65%)	0.030
Diabetes, n (%)	57/151 (37.75%)	32/72 (44.44%)	25/79 (31.65%)	0.077
Smoking, n (%)	17/151 (11.26%)	6/72 (8.33%)	11/79 (13.92%)	0.409
Alcohol, n (%)	13/151 (8.61%)	5/72 (6.94%)	8/79 (10.13%)	0.567
max FJE, mm, median (IQR)	1.16 (2.78)	2.81 (0.963)	0.00 (0.500)	< 0.001
FJE present, n (%)	88/151 (58.28%)	67/72 (93.06%)	21/79 (26.59%)	< 0.001
VAS, median (IQR)	3 (2)	5 (1)	3 (1)	< 0.001

Values are presented as mean ± standard deviation (SD) or median (interquartile range, IQR) for continuous variables, and n (%) for categorical variables. Tests used: independent *t*-test for normally distributed continuous variables; Mann–Whitney U test (MW) for non-normal continuous variables; Pearson’s χ^2^ test or Fisher’s exact test for categorical variables as appropriate. P values two-sided. max FJE: maximum facet joint effusion (mm); LSI: lumbar segmental instability (LSI = 1: unstable; 0: stable). For reproducibility: dataset variable names are max_FJE and instability_bin. BMI: body mass index; FJE: facet joint effusion; LSI: lumbar spine instability; VAS: Visual Analog Scale.

**Table 3:** Multivariable Logistic Regression for Predictors of Lumbar Segmental Instability

Predictor	Coefficient (β)	SE	OR	95% CI	P value
Intercept	−2.863	2.898	-	-	0.323
max FJE (per 1 mm)	1.825	0.268	6.20	3.67–10.48	< 0.001
Sex (male vs. female)	0.047	0.887	1.05	0.18–5.97	0.958
Age (per year)	−0.0078	0.031	0.99	0.93–1.05	0.801
BMI (per kg/m^2^)	0.0102	0.071	1.01	0.88–1.16	0.886
Hypertension (yes vs. no)	0.683	0.624	1.98	0.58–6.72	0.274
Diabetes (yes vs. no)	0.481	0.612	1.62	0.49–5.37	0.432
Smoking (yes vs. no)	−0.925	1.127	0.40	0.04–3.61	0.412
Alcohol (yes vs. no)	0.576	1.157	1.78	0.18–17.19	0.619

Model: multivariable binary logistic regression with dependent variable LSI (unstable = 1, stable = 0). max FJE entered as continuous (mm). OR = exp(β). Model diagnostics performed: assessed linearity in the logit for continuous predictors, multicollinearity (variance inflation factor), goodness-of-fit (Hosmer–Lemeshow), and discrimination (AUC). BMI: body mass index; CI: confidence interval; FJE: facet joint effusion; OR: odds ratio; SE: standard error.

**Table 4:** The Number and Amount of Joint Effusions in Segments With Segmental Instability of Slippage

Number of joint effusions (151)	LSI (72)	Non-LSI (79)	P
Left	66/72	17/79	
Number			
Effusions width (mm)	2.80 ± 0.90	1.31 ± 1.32	< 0.001
Right	66/72	18/79	
Number			
Effusions width (mm)	2.70 ± 0.88	1.20 ± 1.22	< 0.001
Bilateral	67/72	21/79	
Number			
Effusions width (mm)	2.75 ± 0.89	1.25 ± 1.25	< 0.001

LSI: lumbar spine instability.

### Result 2: FJD grading

On the left side, the numbers of facet joints graded 1, 2, and 3 were 21, 58, and 62, respectively. The numbers of facet joints with effusion in these grades were 18, 37, and 28, with effusion sizes of 2.49 ± 0.84, 2.84 ± 1.42, and 2.04 ± 0.76 mm, respectively. One-way analysis of variance (ANOVA) showed a significant difference in mean effusion sizes among the groups (F(2, 80) = 4.051, P = 0.0211).

On the right side, the numbers of facet joints graded 1, 2, and 3 were 34, 62, and 55, respectively. The numbers of facet joints with effusion were 18, 50, and 16, with effusion sizes of 2.07 ± 1.27, 2.73 ± 0.96, and 1.62 ± 1.05 mm, respectively. One-way ANOVA also showed significant differences among groups (F(2, 81) = 8.021, P = 0.0007).

**Figure 5:**
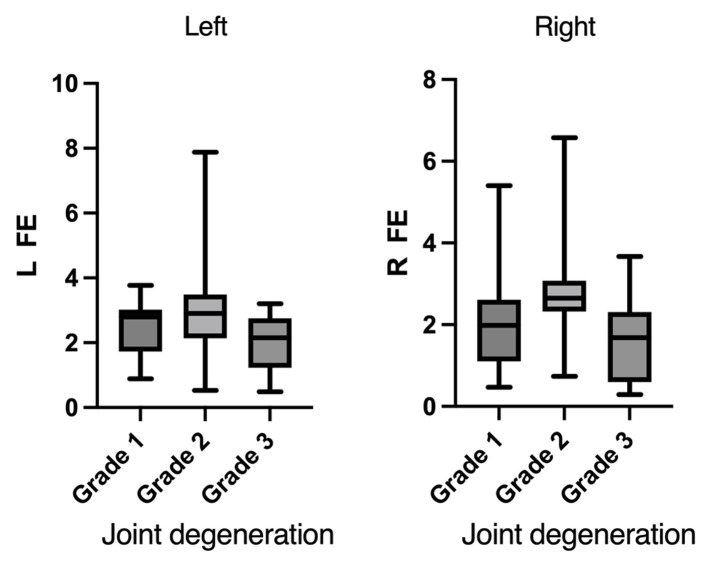
Facet joint effusion (FJE) measurements in patients with lumbar segmental instability, comparing left and right sides with joint degeneration severity.

These findings indicate that for grades 1 and 2, FJE increases with the severity of joint degeneration, while in grade 3, effusion width decreases with advanced FJD (Table 5; Fig. 5).

**Table 5:** Grading of Small Joint Degeneration and Amount of Joint Effusion

Grade	Number of FJE/degenerated joints		Effusions width (mm)	VAS
1	Left	18/21	2.49 ± 0.84	3.77 ± 0.99
	Right	18/34	2.07 ± 1.27	
2	Left	37/58	2.84 ± 1.42	3.86 ± 1.29
	Right	50/62	2.73 ± 0.96	
3	Left	28/62	2.04 ± 0.76	3.44 ± 1.16
	Right	16/55	1.62 ± 1.05	

FJE: facet joint effusion; VAS: Visual Analog Scale.

### Result 3

All patients experienced varying degrees of LBP. The average VAS score for LBP in 88 patients with FJE was 4.30 ± 1.07, while the VAS score in 63 patients without FJE was 2.79 ± 0.68. The VAS scores were significantly higher in patients with FJE compared to those without, with a statistically significant difference between the two groups (P < 0.05).

## Discussion

In our study involving 151 patients with lumbar spondylolisthesis, 67 of 72 patients (93.06%) exhibiting LSI showed FJE, whereas only 21 of 79 patients (26.58%) without instability demonstrated such effusion. This indicates a significant positive correlation between lumbar FJE and dynamic LSI. Notably, among patients with FJE larger than 1 mm, 83.33% had LSI, and this proportion rose to 96.92% in those with effusion exceeding 2 mm. These findings suggest a progressive increase in the probability of dynamic LSI concurrent with the enlargement of FJE. Especially for effusions greater than 2 mm, there is strong evidence implying a high likelihood of instability in DLS segments. High signal intensity of the facet joint on MRI T2-weighted images shows a significant correlation with dynamic LSI, serving as a valuable imaging indicator for potential LSI. The appearance of high signal areas within and around the facet joints on axial T2-weighted MRI images is generally attributed to the presence of fat pads and joint effusion within the facet joint [9]. However, our study further highlights the association between FJE and spinal instability.

The intervertebral discs and facet joints together form a functional spinal unit responsible for maintaining stability and bearing load. Biomechanical studies on cadaveric specimens demonstrate that lumbar facet joints bear approximately 15% of axial load and play a critical role in restricting vertebral motion, thereby protecting the discs from shear forces, excessive flexion, and axial rotational movements [10]. Degeneration of both the intervertebral disc and facet joints can lead to LSI [15, 16]. Currently, spinal instability is primarily assessed by measuring vertebral slippage on standing lateral flexion-extension X-rays. Conventional MRI, performed in the supine position, has limitations in detecting dynamic instability that manifests primarily under load-bearing conditions. Upright MRI, conducted in a standing position, may overcome these shortcomings. For instance, Charest-Morin et al [17] demonstrated that upright MRI more effectively identifies occult spinal stenosis and LSI, though such methods have yet to see widespread clinical adoption. When the patient lies supine during conventional MRI, unstable lumbar segments are unloaded and may reduce posteriorly, causing the formation of a cleft within the degenerated facet joint. Fluid accumulates within this cleft, presenting as a high signal on T2-weighted images, while sagittal MRI views may not reveal overt signs of vertebral slippage. This imaging phenomenon underscores the potential of FJE on axial T2 images to serve as a subtle marker for dynamic LSI, which could otherwise be missed by standard static imaging techniques [8]. Conventional MRI is performed with the patient in the supine position. When lying down, unstable lumbar segments may be reduced due to muscle relaxation, resulting in the formation of clefts within degenerated facet joints. Fluid subsequently accumulates within these fissures, manifesting as high signal intensity on T2-weighted images. Consequently, FJE is more pronounced in cases of LSI [18]. Our study further identified that different patterns of joint effusion distribution carry meaningful implications regarding the progression of lumbar degeneration. Bendersky et al [19] identified large facet effusions—defined as facet effusions greater than 1.5 mm—as a risk indicator for slip instability. Elmose et al [20] demonstrated that even in the absence of vertebral slippage on supine MRI, MRI still has good capability to distinguish instability. Wang et al [21] established a risk prediction model based on a longitudinal study of 220 patients with grade 1 lumbar spondylolisthesis, concluding that facet effusion is an independent risk factor for the progression of lumbar spondylolisthesis. Other researchers have employed different methods to assess risk. For example, Iwata et al [22] analyzed MRI T2 relaxation times as risk factors and found a positive correlation between T2 relaxation times and LSI. Using MRI T2-mapping for novel quantitative assessment of lumbar facet joints may be useful in identifying LSI. Li et al [23] reported that compared with unilateral effusion, bilateral FJEs more likely indicate LSI. Collectively, these studies indicate that FJE signals have a high positive predictive value for LSI. Patients with FJE greater than 1 mm have an approximately eightfold higher likelihood of dynamic spondylolisthesis than those without FJE [24]. However, Even et al [25] found that the presence of FJE has a relatively poor positive predictive value in differentiating dynamic from static lumbar spondylolisthesis. In our study, we found a strong linear correlation between the width of facet joint fluid on MRI and the degree of slippage detected on lateral lumbar X-rays. The presence of facet joint fluid suggests radiological LSI and carries a relatively high positive predictive value. The finding of facet joint fluid on MRI should raise high suspicion for LSI [26, 27]. Compared with Rihn et al (2007), our study extends the evidence by using a larger, multi-level cohort (n = 151, L3–L5 vs. single-level L4–L5), applying direct millimeter quantification of maximal axial facet effusion rather than a ratio index, and providing diagnostic performance metrics (sensitivity, specificity, left/right ROC AUCs). Importantly, we also relate FJE to patient-reported pain (VAS) and describe a non-linear association with facet degeneration grade, which together offer greater clinical context and broader generalizability for using quantitative FJE to screen for dynamic segmental instability [9]. Nonetheless, our data also indicate that MRI should not be used alone to diagnose LSI. Among patients with radiologically confirmed LSI, 6.94% showed no FJE on MRI. Conversely, 23.86% of patients with FJE on MRI did not have radiological evidence of facet joint instability. When significant FJE is identified (exceeding our ROC-derived threshold), clinicians may consider obtaining additional dynamic imaging and integrating imaging findings with the full clinical assessment prior to altering management. Given this, flexion-extension radiographs remain essential for the comprehensive assessment of LSI.

Our studies have shown that the size of FJE is significantly correlated with LBP, but does not show a positive correlation with the degree of FJD. The biomechanics and pathophysiology of degenerative lumbar spine disease are complex and not yet fully understood. The facet joint is a typical synovial joint composed of a joint capsule and hyaline articular cartilage, which covers the subchondral bone of the inferior articular process of the cephalad vertebra and the superior articular process of the caudal vertebra. These joints undergo degenerative changes similar to those seen in other synovial joints affected by osteoarthritis. This degenerative process includes cartilage degradation, subchondral sclerosis, osteophyte formation, and joint effusion [28]. Kirkaldy-Willis [29] divided the spinal degeneration process into three stages: stage 1 is the dysfunction phase of the intervertebral disc and ligament structures, accompanied by minor anatomical changes. Stage 2 is the relative instability phase, characterized by a decrease in disc height, laxity of the joint capsule and ligaments, and joint changes that may lead to abnormal increases in translational and rotational motions. Stage 3 is the restabilization phase, during which progressive degeneration causes increased spinal stiffness, restoring stability through osteophyte formation and fibrosis. Theoretically, during the transition from instability to restabilization, the degeneration process accelerates, and FJE should decrease. At degeneration stage 3, a joint stabilization phase is reached with little FJE [30]. However, there is no necessary correlation between facet joint opening (FJO) observed on CT and FJE seen on MRI [31]. Axelsson and Karlsson [32] found that when disc height decreases by more than 50%, vertebral stability is reestablished. Further analysis of the relationship between bilateral FJE and lumbar stability in patients with effusion revealed a significant correlation, suggesting that lumbar facet joint fluid observed on MRI may indicate the stability of that spinal segment. After instability occurs, physiological loading exceeds the spine’s capacity, exacerbating inflammatory responses. This increases the amount of joint fluid and further promotes fluid accumulation in both facet joints. Spinal instability decreases with increased degeneration of facet joints and intervertebral discs; when FJE diminishes, the spine enters the restabilization phase. Kalichman’s ’s study based on CT scans of a community population showed that the prevalence rates of lumbar facet joint osteoarthritis in age groups < 40, 40–49, 50–59, 60–69, and > 70 years were 24.0%, 44.7%, 74.2%, 89.2%, and 69.2%, respectively [33]. The pain is primarily localized to the neck and lower back, and can also involve the upper or lower limbs, representing referred pain. Lumbar facet joint osteoarthritis pain commonly refers to the buttocks and thighs, rarely extending below the knee [33]. With advancing age, lumbar degeneration worsens, and the prevalence of facet joint osteoarthritis decreases. This may explain why most elderly patients with lumbar spondylolisthesis rarely show FJE on MRI T2-weighted images; smaller FJEs indicate that the affected intervertebral disc has restabilized in DLS [27]. However, some researchers have reported no significant correlation between FJE and low back pain (LBP), cautioning against decisions for spinal fusion based solely on the presence of FJE [34]. Our study showed that FJE in grade 3 FJD was lower than in grades 1 and 2, and the VAS scores for LBP were also reduced correspondingly. These findings indicate that as the lumbar spine restabilizes, the severity of LBP is alleviated to some extent.

After adjustment for demographic and clinical covariates (sex, age, BMI, hypertension, diabetes, smoking, and alcohol), maximum FJE (max FJE) remained strongly associated with radiographic LSI: each 1 mm increase in max FJE was associated with an approximately sixfold increase in the odds of instability (adjusted OR 6.20, 95% CI 3.67–10.48, P < 0.001), supporting the role of FJE as an imaging marker of segmental instability. Nevertheless, our study has several important limitations. First, it is a retrospective, single-center study with a limited sample size and therefore can only identify associations rather than establish causality; these findings require confirmation in larger, prospective, multicenter cohorts [35]. Second, the predominance of L4-level involvement and the higher proportion of female patients reflect consecutive case inclusion at our center rather than selective enrollment. These patterns may be influenced by local care-seeking behavior and regional demographic characteristics (e.g., a relatively larger elderly female population) and therefore may not be generalizable to other settings. Population-based or multicenter studies that incorporate local epidemiology are needed to determine whether these distributions are reproducible. Third, because the prevalence of instability in our sample is relatively high (58.3%), ORs may overestimate the corresponding relative risks; interpretation should emphasize the direction and strength of the association rather than exact risk magnitudes. Fourth, although we adjusted for several potential confounders, the observational design cannot exclude residual confounding by unmeasured factors. Fifth, we assessed only sagittal instability; rotational instability may exist in some segments classified as stable [36]. Sixth, for measurement convenience we used facet joint width on axial MRI as a surrogate for fluid volume—while width and volume are related, this substitution and the three-dimensional anatomy of the joint (axial slices may not always capture the maximal joint space) introduce measurement error. Addressing these limitations and potential sources of bias will be important in future studies and for external validation of our results.

Whether spinal segments with FJE on MRI but without radiographic spondylolisthesis carry a higher risk of future instability remains unclear. If a segment with FJE but no dynamic instability undergoes decompression via laminectomy without fusion, will this result in iatrogenic instability? In cases where LSI has progressed to restabilization due to FJD—with little or no FJE present—and decompression without fusion is performed, is there a risk of re-instability? These questions remain unanswered and may be clarified through careful prospective studies in the future.

Our findings demonstrate a linear correlation between the width of FJE and the degree of instability observed on flexion-extension lumbar radiographs. The presence of FJE on MRI has a relatively high positive predictive value for dynamic LSI. Clinically, when increased FJE is identified on routine lumbar MRI, we recommend obtaining dynamic lumbar radiographs (standing flexion-extension or equivalent) to evaluate for segmental instability; however, the decision to perform fusion or other definitive interventions should be based on integrated clinical and imaging assessment rather than on facet effusion alone.

### Conclusions

In this cohort of 151 patients, millimeter-quantified lumbar FJE on MRI correlated positively with dynamic LSI; the probability of dynamic LSI increased with larger FJE, and segments with FJE > 2 mm had an instability rate of 96.92%. FJE increased as FJD progressed from grade 1 to grade 2, but declined at grade 3, which may reflect late-stage joint collapse, ankylosis, or structural stabilization. Low back pain (VAS) rose with increasing FJE during the degenerative/instability phase and then tended to lessen as FJD advanced to severe (grade 3) degeneration and segments became more stable or fused. These findings support a model in which early-to-moderate degeneration is associated with synovial effusion and increasing instability, while advanced degeneration is associated with reduced effusion and clinical/mechanical stabilization; given the retrospective design, these are associations that require prospective validation.

## Data Availability

The data that support the findings of this study are available from the corresponding author Sun upon reasonable request.
